# Analysis of the Heterogeneous Distribution of Amiloride and Propranolol in Dried Blood Spot by UHPLC-FLD and MALDI-IMS

**DOI:** 10.3390/molecules24234320

**Published:** 2019-11-26

**Authors:** Beatriz Uribe, Oskar González, María Encarnación Blanco, Oihane Elena Albóniga, María Luz Alonso, Rosa María Alonso

**Affiliations:** Analytical Chemistry Department, Faculty of Science and Technology, University of the Basque Country (UPV/EHU), Sarriena s/n, 48940 Leioa, Biscay, Basque Country, Spain; beatriz.uribe@ehu.eus (B.U.); oskar.gonzalezm@ehu.eus (O.G.); sionblanco@gmail.com (M.E.B.); oihaneelena.alboniga@ehu.eus (O.E.A.); marialuz.alonso@ehu.eus (M.L.A.)

**Keywords:** DBS, bioanalysis, UHPLC-FLD, MALDI-IMS

## Abstract

Dried blood spot (DBS) has lately experienced an increase in its use in bioanalysis due to its several advantages compared with traditional blood sampling methods. Nevertheless, the use of DBS with quantitative purposes is hindered by the heterogeneous distribution of some compounds in the supporting matrix and the dependence of the response on different factors, such as the hematocrit, blood volume, and sampling position. In this study the effect of those factors in the analytical response was investigated by ultra high performance liquid chromatography coupled to fluorescence detection, using amiloride and propranolol as model compounds. The results showed a heterogeneous and drug-dependent distribution of the compounds in the blood spot. While amiloride concentration was higher in the center, propranolol concentration was higher in the periphery of the spot. Besides, the influence of the hematocrit on the quantitative results was observed. MALDI mass spectrometry imaging (MALDI-IMS) has allowed study of the distribution of the two cardiovascular drugs when they were placed in the DBS card using water:methanol solutions, demonstrating that they followed a similar distribution pattern as in blood. This work has showed the potentiality of the MALDI-IMS technique to predict the distribution of the drugs in the DBS card.

## 1. Introduction

Dried blood spot (DBS) sampling is a simple technique in which a drop of blood pricked from a heel, ear, or finger [1,2] is placed in a support and is left to air dry prior to analysis. The analyte extraction from the dried blood is usually carried out offline by using different solvents or, alternatively, by employing integrated procedures using online systems [3]. The DBS technique is used as a sampling method for a wide range of analytical techniques, such as DNA-based assays, enzyme activity assays, immunoassays, direct mass spectrometry, and liquid chromatography coupled to different detectors [4].

DBS sampling method was first used with human blood in 1963 by Roberth Gurthrie to detect metabolomic diseases (phenylketonuria) in newborn infants [5]. Since then, it has been employed in many different areas, such as toxicokinetics and pharmacokinetic studies, diagnostic screening, and therapeutic drug monitoring [1,4]. DBS shows many advantages compared with conventional blood, plasma or serum venipuncture collection procedures, such as the stability of the cellulose-fixed analytes, the little blood volume required, the possibility of automation of the sample processing, the easy storage and transportation, or the lower biological risk in comparison with liquid blood samples [2,6,7,8,9]. These benefits, in combination with the improvement of the analytical instrumentation in terms of sensitivity, have resulted in a notable increase in the use of DBS in the last years.

DBS analysis can be performed in two ways: The analysis of the whole blood spot placed in the card or the analysis of a punch of a fixed diameter obtained from the blood spot. The former requires an accurate measurement of the volume placed in the card if a quantitative analysis is to be performed [10,11]. Fortunately, some solutions for its simple measurement have been developed, such as volumetric absorptive microsampling (VAMS) [12]. However, some of these methodologies can be more expensive or require appropriate infractructures in comparison to “traditional” DBS [13]. Therefore the most usual procedure is still based on the analysis of a punch. Nevertheless, there are several factors that influence this approach, i.e., the type of blood (venous or capillary) [9,10], the chemistry of the support [14,15], or the hematocrit (Hct) [16,17,18,19,20,21,22,23,24,25,26]. Among them, Hct is considered the most critical one since it cannot be controlled and varies from one patient to another. The lower the Hct of the blood, the lower its viscosity and, therefore, the broader the blood spot placed in the DBS. Consequently, the amount of blood collected in the punch for low Hct samples will be smaller and the quantification of the analytes will be affected. Even if the influence of the Hct has been deeply studied and some strategies to avoid it or minimize it have been developed [11,16,17,27,28], their implementation remains limited [9,13,22].

Another aspect to be taken into account when performing a quantitative analysis using the punching approach is the distribution of the analytes in the blood spot [29]. Since only a portion of the sample is analyzed, it must be highly representative of the whole. Otherwise, if an area with a lower or a higher concentration of the compound of interest is punched, the quantitative results will be biased. The heterogeneous distribution of some substances has been already reported [15] and a recurring pattern in which the concentration of the analytes is higher in the peripheral area has been identified (the so called “volcano effect” [30]). This fact indicates that the distribution of the analytes should be carefully studied before a DBS method is implemented.

A common approach to study the distribution of the analytes consists of punching the spot at different positions and analyzing them using the intended method. In order to study the distribution in more depth, an alternative approach was applied by Cobb et al. [15] based on the measurement of the radioactivity of radiolabeled compounds. Matrix-assisted laser desorption ionization imaging mass spectrometry (MALDI-IMS) could be of potential applicability, but, so far, DBS is not amenable to this technique without a prior extraction because of the signal suppression related to the blood matrix [3]. Nevertheless, the distribution of the analytes in the card can be studied by MALDI-IMS in matrices other than blood.

In this work, the distribution homogeneity in DBS cards of two antihypertensive drugs with different physicochemical properties, amiloride and propranolol, was studied. The influence of the blood volume, the Hct, and the punching position on the response of the analytes was carefully studied. The analytical method consisted of an optimized extraction procedure prior to the analysis by ultra high performance liquid chromatography coupled to fluorescence detection (UHPLC-FLD). The analysis at different punching positions provided information about the distribution behavior of the drugs that was further investigated by MALDI-IMS.

## 2. Results and Discussion

### 2.1. Model Compounds

**Figure 1:**
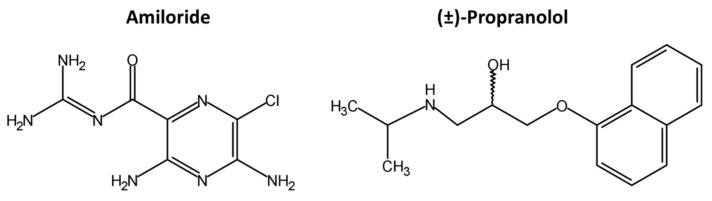
Chemical structures of amiloride and (±)-propranolol.

The study of the distribution of drugs in DBS was performed using two compounds belonging to different families of cardiovascular drugs: Amiloride and propranolol. Amiloride (C_6_H_8_ClN_7_O) is a diuretic with small molecular weight (229.62 g/mol) and low protein binding (<40%). It has two basic groups (pKa: 1.37/5.35), one acidic group (pKa: 11.43), and is moderately polar (logD (pH 7): −0.89). Propranolol (C_16_H_21_NO_2_) is a β-blocker with a single basic group (pKa: 9.67). The molecular weight (259.34 g/mol) is similar to amiloride, the polarity is slightly lower (logD (pH 7): −0.08), and the protein binding is much higher (>90%). The differences in chemical structures between the two molecules can be clearly appreciated in Figure 1.

### 2.2. Sample Treatment and Recovery Calculation

**Figure 2:**
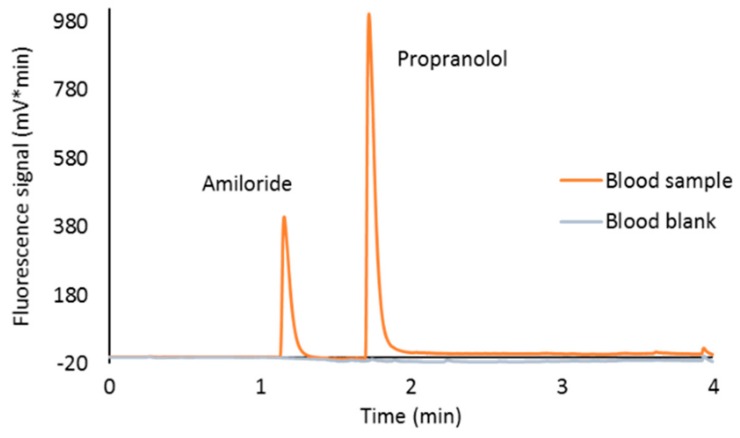
Chromatograms of a blank blood sample and a blood sample spiked at 0.125 mg/L after DBS extraction with 200 µL of methanol:phosphate buffer (pH 2, 0.1 M) (75:25).

Optimal extraction conditions were studied in terms of volume, aqueous phase pH, organic solvent nature (MeOH or ACN), and organic solvent:aqueous phase proportion Analytes showed the best extraction results when using 200 µL of methanol:phosphate buffer (pH 2, 0.1 M) (75:25) solution for a 5.9 mm i.d. punch (taken from the center of the spot) followed by sonication (20 min) and centrifugation (10 min at 4400 rpm). Chromatograms obtained following this procedure for a blank sample and a spiked sample (0.125 mg/L CSF) are shown in Figure 2.

Taking those conditions into account and in order to study if the sample area/extractant volume ratio could affect the recovery, the following procedures were carried out on DBS cards with 25 µL of 40% Hct blood spiked to 1 mg/L of each drug (n = 3):

1. Whole blood spots were extracted using 200 µL of the extractant (the same volume used for punched samples), analyzed, and compared to a standard solution of 0.125 mg/L, which would be the expected concentration if the recovery was 100%.
2. Whole blood spots were extracted using 500 µL of the extractant in order to keep the same sample area/extractant volume ratio used for punched samples, as the area of the whole blood is 2.5 times the area of the punch. These samples were analyzed and compared to a standard solution of 0.05 mg/L.
3. Punched blood spots taken from the center of the drop were extracted using 200 µL of the extractant, analyzed, and compared to a standard solution of 0.05 mg/L, which would be the concentration in the extractant supposing a 100% recovery and a homogeneous distribution of the analyte and the blood in the DBS.

The recovery values for amiloride and propranolol using the three proposed methodologies are gathered in Table 1. The recovery of the analytes when the whole spot was extracted with a 200 µL solution is significantly lower, probably due to the saturation of the extraction solution. Considering that the method was developed for the analysis of a punched sample, the recoveries obtained for the second approach should be more realistic, since the sample area/extractant volume ratio is the same as in the method developed for the analysis of the drugs.

The third extraction method keeps the same sample area/extractant volume but using a punched sample. In this methodology, the final expected concentration was obtained based on the calculation of the blood spot/punch area ratio (measured using AutoCAD2016) and assuming a homogeneous distribution of the drugs. The recovery calculated for propranolol following this approach fits with the one obtained when the whole DBS was extracted with 500 µL of the extraction solution (2). Thus, both methodologies seem comparable for this analyte. On the other hand, the recovery value calculated for amiloride is surprisingly high compared with the approaches in which the whole spot was extracted. This suggests that the assumption of a homogeneous distribution is not correct and the area punched for the analysis (center of the spot) is more concentrated in amiloride than the average of the DBS. This phenomenon could be explained by a heterogeneous distribution of the compound that implies an overestimation of the recovery.

### 2.3. Study of the Distribution of the Analytes in the DBS

#### 2.3.1. Impact of the Hematocrit and Study of the Distribution of the Analyte in DBS Samples by HPLC-FLD

Due to a heterogeneous distribution of the blood or to chromatographic processes of the drugs with the cellulose, the distribution of the analytes can be heterogeneous inside the blood spot [11,12]. Although the mechanisms behind the nonhomogeneous distribution are yet to be understood, it is obvious that under this situation, the hematocrit and the punching position become critical factors for quantitative analysis and should be carefully studied.

In order to study the effect of hematocrit and volume, blood was spiked at 1 mg/L and three different volumes (15, 25, and 35 µL) were assessed (n = 3) for low and high Hct values (25% and 55%). The responses obtained for propranolol showed that the concentration of the analyte was significantly higher in the high Hct samples (Table 2, 95% confidence level). The impact of Hct in the results can be related to the difference in the area of the spot for the same volume of blood: Low Hct samples spread more and, therefore, there is a lower amount of blood in the punch (concentration will be underestimated), while the amount of blood in the punch of the high Hct samples is higher (concentration will be overestimated). Interestingly, no significant differences were observed for amiloride between the results obtained for 25% and 55% Hct samples except for the 15-µL samples. This fact supports the hypothesis of the heterogeneous distribution of amiloride observed during recovery experiments. Although the blood with lower Hct spreads more across the card, if the analyte is prone to remaining in the center of the spot, the effect of the Hct will become less apparent.

In order to confirm that the differences in propranolol response were related to the spread of the blood, a simple correction of the Hct effect was assessed by multiplying the chromatographic response by the surface area squared. After correction, no significant difference was observed (Table 2), which indicates that the response is indirectly correlated with the surface of the drop. Not surprisingly, this compensation had a negative effect in amiloride, since the chromatographic response showed no dependency with Hct.

The distribution of the analytes was studied in a more exhaustive way in the 35-µL samples at three specific positions (central, upper peripheral, and lower peripheral) using small punches (1.2 mm i.d.). Samples in the periphery were punched both from the upper and the lower region to study if the fact that the paper is not completely horizontal during the drying step affects the distribution. The sampling was carried out in triplicate by taking five small punches from each area. The small punches taken from each area were extracted simultaneously to guarantee a response above the quantitation limit of the method.

**Figure 3:**
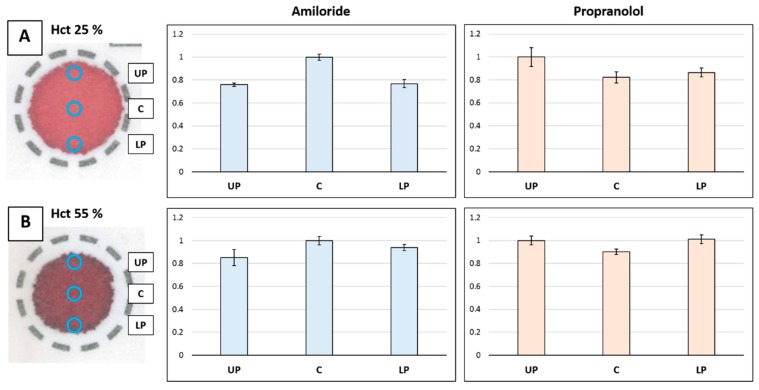
Normalized chromatographic responses of the analytes belonging to central (C), upper peripheral (UP), and lower peripheral (LP) zones of the blood spot at low hematocrit (25%, (**A**)) and high hematocrit (55%, (**B**)) levels. Error bars show the standard deviation.

The response obtained for each analyte after the analysis of the three different positions for low Hct (25%) and high Hct (55%) can be observed in Figure 3. Results were normalized to the more intense response in order to facilitate data interpretation. A clear difference in the analytes’ distribution behavior was noticed when comparing the response of the samples obtained in the center and in the periphery. In order to easily indicate the distribution shape of drugs, de Vries et al. [24] defined the central/peripheral (C/P) ratio value as the division of the response obtained in the central zone (C) by the one obtained in the peripheral zone (P). In this way, it is possible to show if the drug is more concentrated in the center (C/P > 1), in the periphery (C/P < 1), or if it is homogeneous (C/P ~ 1). In our study, after calculating the average of the two peripheral areas, the C/P values for amiloride were 1.31 and 1.12 for the 25% and 55% Hct samples, respectively. For propranolol, the C/P ratios were 0.88 and 0.89.

As it can be observed, the amiloride concentration was higher in the central zone, which explains why the recovery calculated when punching in that area was overestimated (Table 1). Interestingly, the distribution homogeneity is higher at the 55% Hct level, most likely influenced by the smaller size of the drop and a closer position of the different punching regions. Regarding propranolol, it showed higher concentration values at the peripheral zone, which fits with the volcano effect already reported for other drugs. It is believed that this phenomenon is due to the fact that the drug migrates with the blood to the perimeter, although chromatographic mechanisms can also be involved. De Vries et al. [19] studied the distribution of 12 different compounds in four different card types and observed that many of them tend to migrate to the perimeter as propranolol does. They observed that the distribution was compound dependent and although they could not establish a clear trend based on the polarity or protein binding capacity, it is remarkable that the most polar analyte they studied behaved differently to the more non-polar ones. That could also explain the distribution pattern of amiloride. Taking into consideration that it is a very polar molecule with seven nitrogen groups and a low protein binding, it may strongly interact with the cellulose in the card, and in consequence not easily migrate with the blood. Indeed, the lower recoveries obtained for amiloride compared to propranolol when the whole DBS was analyzed point out a stronger interaction of cellulose with amiloride. In any case, both the heterogeneous distribution of amiloride and propranolol show the importance of studying the analyte distribution when a DBS method is developed.

#### 2.3.2. Study of the Distribution of the Analytes in the DBS Support by MALDI-IMS

Although to date direct analysis of DBS by MALDI-IMS has not been successfully applied because of the matrix effect, this technique allows study of the analyte distribution in the DBS cellulose support replacing blood with solvents, such as water or methanol. For this aim, three different solutions containing amiloride and propranolol at 5 mg/L in 2:98, 50:50, and 100:0 methanol:water proportions were prepared. In total, 20 µL of these solutions were placed in DBS cards and left to air dry. After CHCA spraying and MALDI-IMS analysis, images of amiloride and propranolol were extracted.

**Figure 4:**
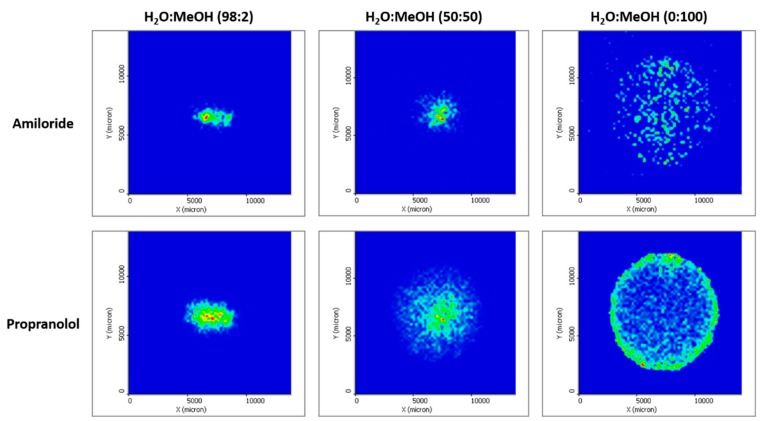
Different distribution of amiloride and propranol observed by MALDI-IMS after a 20-µL drop of different combinations of H_2_O:MeOH was placed in the cellulose support.

The distribution of amiloride and propranolol in the DBS card after applying the sample using different solutions can be observed in Figure 4. It must be considered that it is not only solvent–analyte and analyte–cellulose interactions explain the distribution of the compounds. Due to the different superficial tension of the solvents, the drop will expand more when the amount of methanol is higher. Therefore, the interaction of the solution with the support should also be taken into account. That is the reason why when the solvent is mostly water, both analytes are concentrated in the center of the DBS. The support absorbs the solution before it has time to spread in the card. Nevertheless, it can be observed how propranolol expansion is slightly broader.

When the methanol amount is increased, the chromatographic behavior of the molecules is more easily perceived. In a water:methanol (50:50) solution, propranolol migrates significantly more than amiloride. The reason behind this distribution is attributed to a paper chromatography-like process between the stationary phase (cellulose) and the mobile phase (solvent). In paper chromatography, a separation mechanism is believed to happen between a layer of water attached to cellulose and the mobile phase. Amiloride, a very polar molecule with several N groups that are able to create hydrogen bonds, has stronger interactions with the stationary phase and therefore migrates less when the drop is spreading. Propranolol, on the contrary, seems to migrate easier, probably due to its higher affinity with methanol. This fact can be clearly observed in the solutions containing 100% methanol, where the propranolol is located in the perimeter of the drop, a distribution that matches with the volcano effect observed in the blood samples.

## 3. Materials and Methods

### 3.1. Reagents and Solutions

Amiloride hydrochloride and (±)-propranolol hydrochloride were purchased from Sigma-Aldrich (St. Louis, MI, USA). HPLC-grade methanol used for the preparation of stock and working solutions and the HPLC grade acetonitrile used for the mobile phase was provided by Romil (Cambridge, England). For the preparation of the extraction buffer solution, potassium dihydrogen phosphate (>99%) obtained from Merck (Darmstad, Germany) and phosphoric acid (85%) supplied by Panreac (Barcelona, Spain) were used. Ultrapure analytical water was obtained from a Milli-Q Element A10 system (Millipore, Milford, CT, USA). α-cyano-4-hydroxycinnamic acid (CHCA) and DAN (1,5-Diaminonaphthalene) from Sigma-Aldrich and DHB (2,5-Dihydroxybenzoic acid) supplied by Acros Organics (Geel, Belgium) were used as ionizable matrices for MALDI-IMS experiments. Standard stock solutions of 1000 mg/L were prepared in methanol for each analyte separately. With those solutions, 50 mg/L working solutions in methanol were prepared.

### 3.2. Instrumentation

The DBS cards used were Protein saver 903 of Whatman (Florham Park, NJ, USA). A regular office puncher with a diameter of 5.9 mm, and a 1.2-mm puncher purchased from Harris (CA, USA) were used to collect the samples. Analyses were performed on an Acquity Ultra Performance Liquid Chromatography (UPLC) system (Waters, Milford, CT, USA) coupled to a FLD detector. The chromatographic column used was an Acquity BEH C18 (2.1 × 50 mm, 1.7 µm) from Waters. System control, data collection, and data processing were accomplished using Empower 2 software (version 6.20) from Waters. The statistical treatment of the results was carried out using Microsoft Excel 2010 (Redmond, Washington, DC, USA). AutoCAD2016 software (AUTODESK, San Rafael, CA, USA) was used for measuring the surface of the blood drops in the DBS. The pH was measured with a Crison GPL22 pH-meter (Barcelona, Spain).

For MALDI (Matrix Assisted Laser Desorption) analysis, a MALDI LTQ 25 Orbitrap XL (ThermoFisher, CA, USA), equipped with an N2 laser (LTB, Berlin, Germany, model MNL 100, 100 μJ max power, elliptical spot, 60 Hz repetition rate), was used. The matrix was deposited with the aid of a HTX TM-Sprayer (HTX Technologies, NC, USA). Data treatment was performed using Thermo ImageQuest.

### 3.3. Blood Samples

Blood samples covering the range of 22% to 55% Hct values were generously provided by Hematology service of University Hospital of Basurto (Bilbao, Spain) previous ethical consent of patients. Due to the impossibility of access to fresh blood whenever necessary, the samples obtained were storage in the freezer (−80 °C). The frozen blood samples were thawed for being spiked with the analytes and deposited in the DBS support. This was done under blood heating conditions to a temperature of 37 °C to simulate real sampling conditions. Blood samples were spiked with working solutions to a concentration of 1 mg/L, and the volume required for each study was placed in the support and left to dry at room temperature for two hours.

### 3.4. Chromatographic Conditions

Chromatography was performed using a 0.01% formic acid solution as the aqueous mobile phase (A) and acetonitrile as the organic modifier (B) at a flow rate of 0.55 mL/min. The total run time was 6 min with an elution gradient as follows: 0–0.5 min, 1% B; 0.5–3 min, linear change from 1% to 95% B; 3–3.5 min, 95% B; 3.5–3.6 min, from 95% to 1% B; 3.6–6 min, 1% B. During the chromatographic analysis, the column was thermostated at 35 °C and samples were kept at 10 °C in the autosampler. The excitation/emission wavelengths of the FLD detector were 363/415 nm for amiloride and 287/340 nm for propranolol.

### 3.5. MALDI-IMS Analysis Conditions

The optimal MALDI conditions for the analytes were fixed after a preliminary study. The best ionizable matrix was choosen between CHCA, DAN, and DHB, and the appropriate deposition technique (spraying or sublimation). Afterwards, the laser energy was fixed among 40, 50, and 60 KJ. MALDI experiments were performed using CHCA (10 mg/mL) in acetonitrile:H_2_O (70:30). The matrix deposition was carried out using the HTX sprayer at a block temperature of 75 °C. The spraying device was moved at a speed of 1200 mm/min. The pattern was adjusted with a line spacing of 3 mm and 10 passes were carried out in order to apply a homogeneous matrix layer. The matrix flow rate of the pump was 60 μL/min and the air pressure was set to 10 psi. The nozzle to DBS distance of the sprayer was set to 46 mm. In total, 50 KJ laser energy was used for the ionization of the samples. A mass resolution of 30,000 was used to record the data and the scanning range was 220–470 m/z. After the analysis, images of amiloride (*m*/*z* 230.0546) and propranolol (*m*/*z* 260.1668) were extracted.

## 4. Conclusions

In this work, the heterogeneous distribution of amiloride and propranolol in DBS was observed by UHPLC-FLD. Moreover, an important impact of hematocrit in the results was reported, although it was corrected in some extent, as, interestingly, the drugs follow different distribution patterns: Amiloride is more concentrated in the central area of the blood drop while propranolol is more concentrated in the peripheral area. The disparate behavior was connected with the migration of the blood and chromatographic interactions with the support. We studied the analyte distribution with different solvents by MALDI-IMS and observed that the patterns obtained when the analytes were placed in the support using methanol fit with the ones obtained for the DBS by UHPLC-FLD. This is, to our knowledge, the first time MALDI has been used to study the distribution of drugs in DBS cards. Obviously, it must be considered that solvent–analyte interactions cannot be compared with blood–analyte interactions (including protein binding). Thus, these experiments do not try to mimic the behavior of the analyte in the matrix but to better understand the analyte dispersion. In this sense, MALDI-IMS can be a simple and smart way to predict the distribution in a visual way.

The effect of Hct in the results could also be avoided by analyzing the whole blood drop, but, from the point of view of a reliable quantitation, all the blood drops (calibration and study samples) should have the same volume. Although that is not a problem for samples prepared in a laboratory, it would make sampling more complicated under other conditions (in resource-limited settings, hospitals, or clinical laboratories, where they do not have state-of-the-art techniques for DBS sampling) [9,10].

## Figures and Tables

**Table 1:** Recoveries (mean value (n = 3) ± standard deviation) for amiloride and propranolol following three different procedures. Blood drops (40% Hct) of 25 µL spiked to 1 mg/L were used.

	Amiloride (%)	Propranolol (%)
(1) Whole spot +200 µL extractant	60 ± 2	77 ± 3
(2) Whole spot + 500 µL extractant	70 ± 1	88 ± 2
(3) Punch + 200 µL extractant	95 ± 3	85 ± 7

**Table 2:** Statistical *p*-values obtained for amiloride and propranolol different samples before and after area correction.

Analyte	Volume (µL)	*p-*Value (Before Area Correction)	*p-*Value (After Area Correction)
Amiloride	15	0.00917	0.000186
	25	0.33358	0.042743
	35	0.10356	0.000010
Propranolol	15	0.001917	0.079917
	25	0.000200	0.589000
	35	0.000009	0.546900
